# Multi-omics insights into immune dysregulation and candidate biological programs in kawasaki disease: toward precision immunomodulation

**DOI:** 10.3389/fped.2026.1960681

**Published:** 2026-10-12

**Authors:** Yun Han, Jiaying Liu, Chengxu Song, Li Fu, Yu Gu, Yu Zeng, Zhenzhuang Zou

**Affiliations:** 1 Department of Pediatrics, The Fifth Affiliated Hospital of Zunyi Medical University, Zhuhai, Guangdong, China; 2 Department of Pediatrics, Shenzhen Futian District Maternity & Child Healthcare Hospital, Shenzhen, Guangdong, China

**Keywords:** biomarkers, coronary risk, immune dysregulation, kawasaki disease, multi-omics, precision immunomodulation, single-cell sequencing

## Abstract

Kawasaki disease (KD) is an immune-mediated pediatric vasculitis characterized by dynamic interactions among genetic susceptibility, innate and adaptive immune activation, endothelial dysfunction, and vascular remodeling. Although intravenous immunoglobulin (IVIG) therapy substantially reduces coronary complications, 10%–20% of patients show persistent or recurrent fever after initial IVIG treatment, and reliable clinically validated molecular approaches for diagnosis, treatment-response prediction, and coronary-risk stratification remain unavailable. Recent advances in multi-omics technologies, including genomics, epigenomics, transcriptomics, single-cell sequencing, proteomics, metabolomics, and microbiome profiling, have expanded our understanding of KD-associated immune dysregulation. However, the clinical interpretation of these molecular findings remains challenging because of limited cohort sizes, heterogeneous control populations, treatment-related confounding, and insufficient external validation. In this review, we integrate evidence across multiple molecular layers and propose a framework in which current KD multi-omics findings converge into candidate biological programs rather than fixed molecular subtypes. These programs may represent temporally dynamic biological states along a shared inflammatory trajectory, influenced by disease phase, cellular composition, genetic background, and treatment exposure. Synthesized from convergent multi-omics evidence rather than derived from formal molecular classification, these candidate biological programs include myeloid inflammatory activation characterized by S100A8/A9 and IL-1/NF-*κ*B signaling, interferon-associated immune responses, and vascular injury/remodeling responses involving endothelial activation and extracellular matrix regulation. Current evidence supports biologically meaningful molecular programs that may contribute to clinical heterogeneity, but prospective validation is required before molecular signatures can be applied as clinical classifiers or treatment-selection tools. Integrating multi-omics approaches with functional validation, longitudinal sampling, and multicenter studies may facilitate the development of precision immunomodulation strategies for Kawasaki disease.

## 1 Introduction

Kawasaki disease (KD) is an acute systemic vasculitis that predominantly affects infants and young children. Since its first description by Tomisaku Kawasaki in 1967, KD has become recognized as the leading cause of acquired heart disease among children in many developed countries (1, 2). The disease primarily involves medium-sized arteries, particularly coronary arteries, and may result in coronary artery aneurysms (CAAs), myocardial ischemia, thrombosis, and long-term cardiovascular complications (3, 4).

The incidence and clinical characteristics of KD vary substantially among different ethnic populations. The highest incidence has been reported in East Asian countries, particularly Japan, Korea, and Taiwan, suggesting an important contribution of genetic susceptibility (5–7). Epidemiological studies and familial aggregation analyses further support the concept that KD results from complex interactions between inherited genetic factors and environmental triggers (8, 9).

Although the precise etiology of KD remains unknown, increasing evidence suggests that abnormal activation of both innate and adaptive immunity plays a central role in disease development. Infectious or environmental stimuli may activate pattern-recognition receptor pathways, resulting in excessive production of inflammatory mediators, recruitment of immune cells, endothelial activation, and vascular injury (10, 11). KD diagnosis and treatment remain grounded in clinical features, echocardiography, and conventional laboratory measures. Multi-omics may refine this framework, but the available evidence supports overlapping molecular programs rather than established patient classes.

However, incomplete KD, especially in infants, often lacks typical clinical manifestations, resulting in diagnostic delay. Conventional inflammatory markers, including C-reactive protein (CRP), erythrocyte sedimentation rate (ESR), and white blood cell count, are useful indicators of systemic inflammation but lack sufficient specificity for early diagnosis and prognosis prediction. Intravenous immunoglobulin (IVIG) combined with aspirin remains the standard treatment for KD. Early administration of IVIG significantly decreases the incidence of coronary artery lesions (12, 13). Nevertheless, approximately 10%–20% of children exhibit inadequate response to initial IVIG therapy and remain at increased risk of coronary complications (14, 15). Existing clinical scoring systems, including the Kobayashi, Egami, and Sano scores, have limited predictive performance across different ethnic populations, emphasizing the need for molecular biomarkers that can accurately identify high-risk patients (16–18). High-throughput sequencing has enabled systematic characterization of disease-associated molecular alterations (19–21). In parallel, transcriptomic approaches have revealed reproducible gene-expression signatures associated with acute inflammation, immune-cell activation, and vascular injury (22, 23).

Recent transcriptome meta-analysis studies integrating human and animal KD datasets have further demonstrated conserved molecular changes involving genes such as S100A8, S100A9, CD14, CD74, IFITM2, and IFITM3, highlighting the importance of innate immune activation and interferon-related pathways in KD pathogenesis (24). More recently, single-cell RNA sequencing (scRNA-seq) has provided higher-resolution views of monocyte, lymphocyte, and other PBMC states in KD. These studies complement, but do not replace, whole-blood and granulocyte-focused analyses of neutrophil biology. The emerging picture is not one of fixed molecular subtypes, but of immune and vascular programs whose prominence changes with disease phase, treatment exposure, and the tissue or cell population sampled. Integration of genomics, epigenomics, transcriptomics, proteomics, metabolomics, and microbiome data provides a systems-level framework for connecting genetic susceptibility with dynamic immune states, vascular responses, and clinical heterogeneity (Figure 1) (25–27).

**Figure 1:**
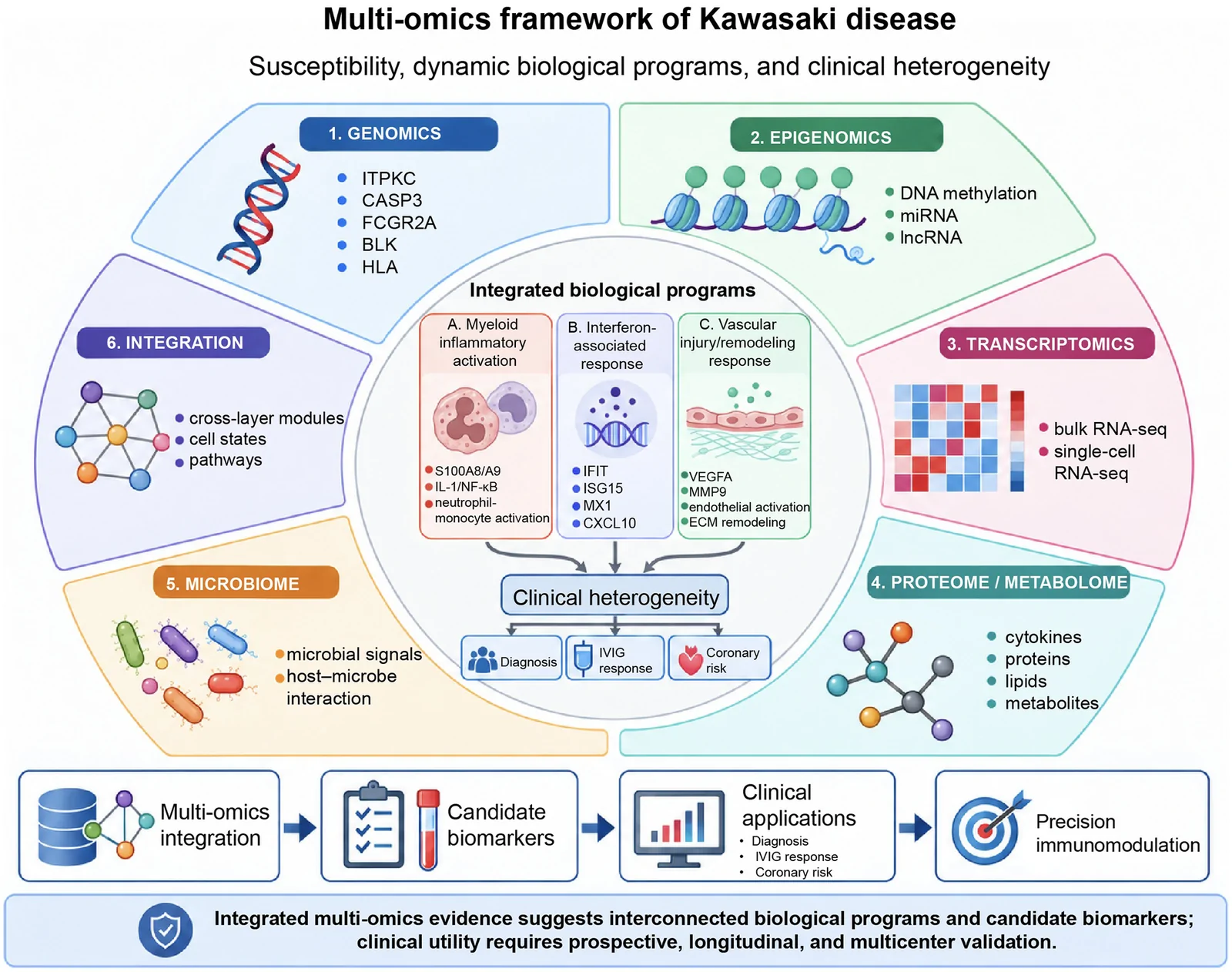
Multi-omics framework of kawasaki disease immune dysregulation and clinical heterogeneity.

**Figure 2:**
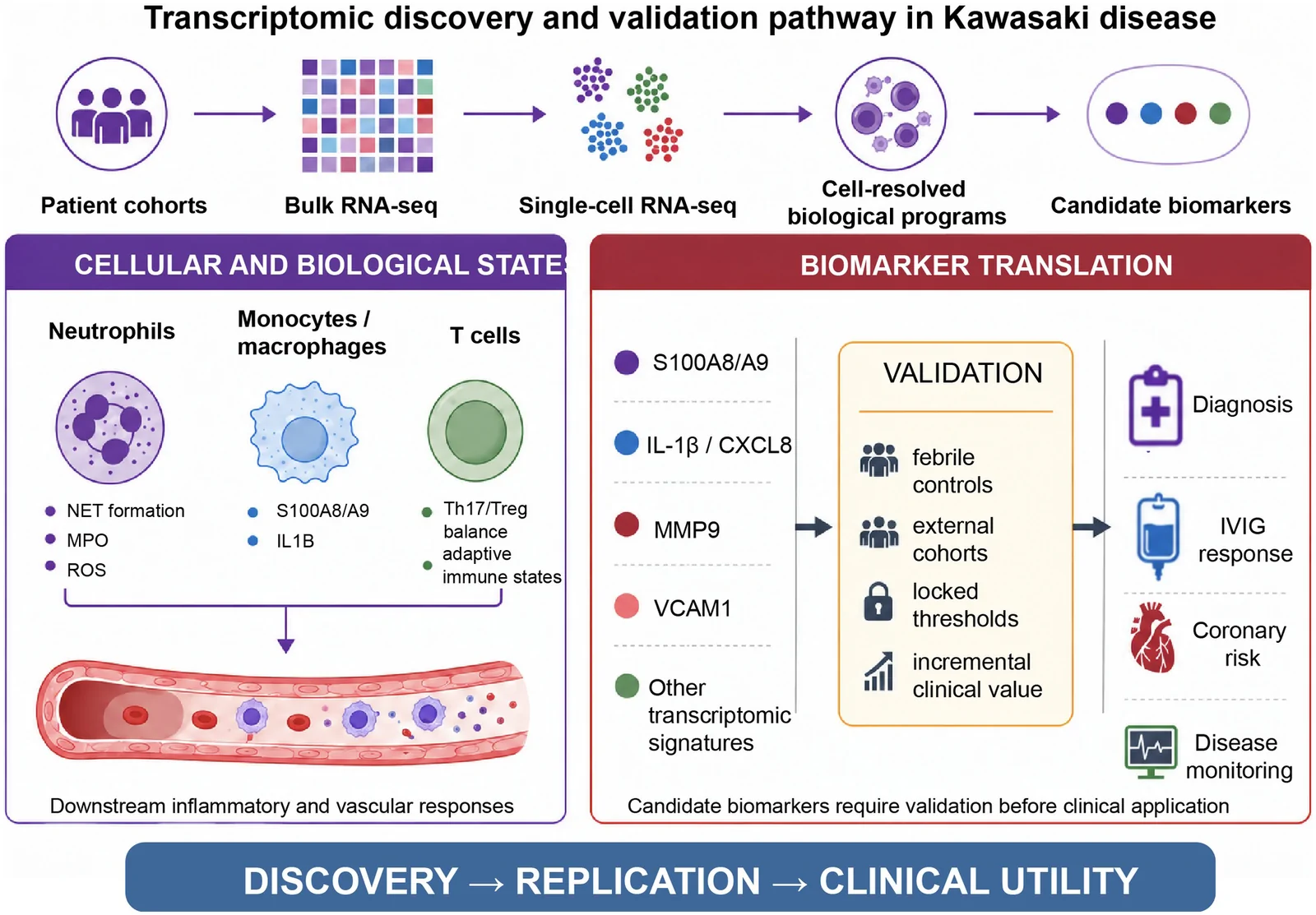
Transcriptomic discovery and validation pathway. Bulk and single-cell studies resolve pathway- and cell-state signals; conventional PBMC datasets underrepresent mature neutrophils. Candidate markers must be tested with febrile controls, independent cohorts, locked thresholds, calibration, and incremental clinical value before use in diagnosis, IVIG-response prediction, or coronary-risk assessment.

This review aims to move beyond a catalogue of molecular alterations by integrating multi-omics evidence into a conceptual framework of immune dysregulation and candidate biological programs in Kawasaki disease. We systematically evaluate evidence from genomics, epigenomics, transcriptomics, single-cell sequencing, proteomics, metabolomics, and microbiome studies, while distinguishing reproducible biological programs from exploratory signals. We further discuss how inflammatory, interferon-associated, and vascular-remodeling programs may contribute to clinical heterogeneity and provide a rationale for future biomarker-guided precision immunomodulation. Unlike previous reviews focusing mainly on individual omics layers or specific molecular biomarkers, this review emphasizes the distinction between biological programs and molecular endotypes, integrating evidence maturity, temporal dynamics, and translational requirements. Importantly, current molecular patterns are considered research frameworks requiring prospective validation rather than established diagnostic or therapeutic classifiers. The principal candidate biomarker classes, evidence maturity, intended clinical uses, and validation gaps are summarized in Table 1.

**Table 1:** Candidate multi-omics biomarkers in kawasaki disease: evidence, clinical implications, and limitations.

Candidate biomarker or signature	Biological program	Evidence maturity	Intended clinical use	Specificity limitation	Key validation gap	Representative evidence
S100A8/A9 (calprotectin)	Neutrophil/monocyte alarmins; TLR4-linked innate inflammatory activation	Moderate—replicated across transcript and protein studies	Adjunctive diagnosis and inflammatory-activity assessment	Also elevated in bacterial infection, viral infection, and other inflammatory disorders	Limited specificity vs. febrile controls; no standardized clinical threshold	(22, 24, 28–31)
IL-1/NF-*κ*B-related inflammatory signature	Inflammasome-associated cytokine activation, myeloid inflammation, and endothelial inflammatory signaling	Moderate—supported by mechanistic and cross-platform evidence	Pretreatment inflammatory-risk enrichment and research stratification for targeted therapy trials	Represents a shared inflammatory pathway rather than KD-specific biology	No standardized assay; limited prospective validation for treatment selection	(11, 23, 32–34)
Inflammasome-associated cytokine signaling (IL-1 family, including IL-18-related pathways)	NLRP3 inflammasome activation; IL-1 family-mediated inflammatory amplification	Low–moderate—mechanistically supported but heterogeneous across cohorts	Research stratification of inflammatory intensity and potential pathway-directed therapy studies	Inflammasome activation occurs in multiple inflammatory diseases	Limited prospective validation and unclear predictive value for treatment response	(11, 32–34)
IL-6-centered inflammatory signature	Cytokine amplification and systemic inflammatory activation	Moderate—biologically supported but heterogeneous across cohorts	Inflammatory burden assessment and potential IVIG-response risk stratification	IL-6 elevation occurs in multiple infectious and inflammatory diseases	Lack of standardized measurement strategy and prospective treatment-response validation	(11, 35)
Neutrophil activation signature (FCGR3B, CSF3R, MMP8)	Granulocyte expansion, degranulation, and matrix injury	Moderate—recurrent whole-blood transcriptomic signal	Adjunctive diagnosis and inflammatory-burden assessment	Strongly influenced by leukocyte composition and infection-related inflammation	Cellular composition dependence; limited external validation across populations	(22, 24, 30, 36)
Interferon-stimulated gene signature (IFIT/IFITM, ISG15, MX1, CXCL10)	Interferon-associated immune activation program	Low–moderate—reproducible in some cohorts but variable	Research stratification of interferon-enriched immune states	Shared with viral infection and antiviral immune responses	Uncertain temporal stability and disease specificity; requires validation with febrile viral controls	(22, 23, 34)
MMP9-centered vascular-remodeling signature	Extracellular-matrix degradation and vascular remodeling	Moderate—supported by vascular pathology and molecular studies	Component of coronary-risk assessment models	May reflect general vascular injury rather than KD-specific coronary pathology	No prospective cutoff; circulating markers may not fully represent coronary tissue changes	(36–40)
Endothelial activation markers (VEGFA, ICAM1, VCAM1)	Endothelial activation, vascular permeability, and leukocyte adhesion	Low–moderate—biologically plausible but heterogeneous	Research models for vascular injury and coronary-risk assessment	Endothelial activation is common in systemic inflammation and infection	Variable outcome definitions, sampling timing, and limited longitudinal validation	(36–40)
Complement/coagulation-related protein signature (e.g., FGG, C1QC)	Complement activation, coagulation regulation, and inflammatory vascular responses	Low–moderate—mainly exploratory proteomic evidence	Coronary-risk assessment and inflammatory-state characterization	May represent systemic inflammation or endothelial injury rather than KD-specific mechanisms	Small discovery cohorts and limited external validation	(41, 42)
NT-proBNP and cardiac stress-associated markers	Myocardial stress, cardiac involvement, and inflammation-associated cardiovascular response	Moderate—supported by prospective clinical studies and meta-analytic evidence	Assessment of cardiac involvement and adjunctive coronary-risk stratification	Elevated NT-proBNP may occur in other cardiac or systemic inflammatory conditions	Need standardized thresholds and prospective validation of incremental value beyond echocardiography and conventional inflammatory markers	(2, 43, 44)
Non-coding RNA panels, including miR-223	Granulocyte regulation and vascular-cell signaling	Low—small cohorts and assay heterogeneity	Minimally invasive candidate diagnostic panel	Influenced by age, cellular composition, inflammation, and technical normalization	Lack of standardized assays and external validation	(45–47)
Proteomic/metabolic panels	Secreted inflammatory proteins, complement/coagulation factors, lipid metabolism, and bile-acid pathways	Low—exploratory multi-platform evidence	Multianalyte diagnosis, IVIG-response risk stratification, or coronary-risk research panels	Reflect systemic inflammatory and metabolic states that may occur in other diseases	Platform effects, pre-analytical variation, dietary/medication effects, and limited multicenter validation	(30, 41, 42, 48)

All listed biomarkers and signatures represent candidate molecular or clinical markers under investigation. None has established prospective clinical utility as a stand-alone diagnostic, treatment-selection, or coronary-risk prediction tool. Validation against clinically relevant febrile controls, standardized clinical outcomes, and external multicenter cohorts is required before clinical application.

Compared with previous reviews that primarily focused on genetic susceptibility, immune mechanisms, individual omics approaches, or specific molecular biomarkers, this review provides an integrative framework linking multi-omics-derived signals with biological programs, clinical decision points, and translational requirements. Rather than proposing definitive molecular classifications, we emphasize that current KD signatures represent candidate biological programs shaped by disease phase, cellular context, and treatment exposure. In addition, we systematically evaluate the evidence maturity, clinical applicability, and major validation barriers of candidate biomarkers across three clinically relevant domains: diagnosis, IVIG-response prediction, and coronary artery risk stratification. This perspective aims to bridge molecular discovery with future precision immunomodulation strategies in KD.

Genetic susceptibility and epigenomic regulation interact with dynamic transcriptomic, proteomic, metabolomic, and microbiome-associated signals to shape interconnected biological programs, including myeloid inflammatory activation, interferon-associated immune responses, and vascular injury/remodeling responses. These programs represent overlapping biological states rather than validated molecular subtypes. Current evidence supports their association with different aspects of clinical heterogeneity, including inflammatory persistence, IVIG response and treatment-related outcomes, and coronary outcomes; however, none of these programs currently represents an established molecular subtype or validated causal mechanism.

## 2 Genetic susceptibility defines the immune landscape of Kawasaki disease

### 2.1 Evidence supporting genetic susceptibility

Genetic susceptibility represents the upstream component of KD immune dysregulation and is supported by three complementary observations: ancestry-related differences in incidence, familial aggregation, and reproducible genetic association signals. The markedly higher incidence among East Asian populations and increased risk among affected families indicate that inherited factors contribute to disease susceptibility (5, 6, 49). However, genetic variants identified by GWAS and candidate-gene studies generally exert modest effects and are insufficient to explain the dynamic inflammatory phenotypes observed during acute disease (19–21). Instead, genetic susceptibility may establish a permissive immune background upon which environmental triggers initiate downstream inflammatory and vascular responses.

### 2.2 Genetic pathways shaping immune activation in Kawasaki disease

Genome-wide association studies and functional investigations have identified several immune-regulatory pathways contributing to KD susceptibility, including calcium–NFAT signaling (ITPKC and CASP3), Fc-receptor-mediated immune-complex regulation (FCGR2A), B-cell co-stimulation (BLK and CD40), and antigen presentation pathways involving HLA regions (19, 20, 50). Rather than representing independent disease determinants, these genetic loci may influence immune activation thresholds, antigen recognition, and inflammatory responsiveness, thereby shaping individual susceptibility to exaggerated immune activation after environmental triggers.

The strength of evidence varies among identified loci. FCGR2A, BLK/CD40, and HLA associations have been supported by genome-wide analyses, whereas the biological interpretation of ITPKC and CASP3 has been further informed by functional and mechanistic studies (19–21, 50–53). However, the contribution of these variants to disease phenotype, coronary artery complications, or treatment response remains incompletely defined. Replication has been strongest in East Asian populations, while differences in ancestry, phenotype definitions, sampling strategies, and coronary outcome assessment limit direct comparison among studies. Therefore, current genetic findings provide an important framework for understanding immune susceptibility in KD but are unlikely to independently define clinically actionable molecular subtypes. Table 3 summarizes the major susceptibility loci according to biological pathway, evidence strength, and potential clinical interpretation.

### 2.3 Genetic factors and coronary artery complications

Coronary artery complications represent a complex downstream phenotype resulting from interactions among inherited susceptibility, immune activation, endothelial dysfunction, and vascular remodeling. Compared with genetic determinants of KD susceptibility, the evidence linking specific variants to coronary outcomes remains less consistent. Studies involving VEGFA-related pathways and matrix metalloproteinases (MMPs) suggest that genetic variation may influence endothelial permeability, extracellular-matrix turnover, and vascular injury responses during acute inflammation (36–39, 45, 54, 55).

However, current evidence should be interpreted cautiously, as some studies integrate genetic variants, transcript abundance, and circulating protein levels without clearly distinguishing different biological layers. These findings support a potential contribution of vascular-remodeling pathways to coronary pathology but have not yet established a reproducible genetic predictor for coronary artery aneurysm development. Future studies integrating genomic information with transcriptomic, proteomic, and clinical variables may better capture the multifactorial nature of coronary risk in KD.

### 2.4 Genetic determinants of IVIG resistance and treatment-response heterogeneity

Genetic studies have also explored whether inherited variants contribute to heterogeneity in response to intravenous immunoglobulin (IVIG) therapy. Associations involving ITPKC, FCGR2A, and CASP3 have been investigated in relation to IVIG responsiveness; however, findings remain inconsistent across populations and are generally weaker than evidence supporting disease susceptibility (14, 19, 20, 51, 56).

To date, no individual variant or polygenic score has demonstrated sufficient discrimination, calibration, and ancestry transportability to guide treatment selection. Rather than acting as independent determinants of therapeutic response, genetic factors may represent one component of a broader immune-risk landscape that interacts with inflammatory state, disease phase, and vascular involvement.

## 3 Transcriptomic evidence reveals candidate biological programs in Kawasaki disease

### 3.1 Overview of transcriptomic approaches

Although genetic studies have identified inherited susceptibility factors associated with KD, genetic variation alone cannot explain the dynamic inflammatory responses and heterogeneous clinical manifestations observed during the acute phase of disease. Transcriptomic approaches provide a complementary perspective by capturing disease-associated changes in gene expression and immune-cell states across different biological contexts, including circulating immune cells and vascular tissues (22, 23). Early transcriptomic investigations of KD primarily relied on microarray-based approaches and revealed coordinated alterations in inflammatory pathways and immune activation signatures. With the advancement of RNA sequencing technologies, transcriptomic analyses have progressed from bulk population-level profiling toward high-resolution single-cell approaches, enabling characterization of cell-type-specific expression patterns, immune-cell states, and potential intercellular regulatory networks. Recent single-cell transcriptomic and integrative multi-omics studies have further refined this framework by resolving immune-cell states, cellular interactions, and regulatory networks at higher resolution. However, most available KD single-cell datasets remain limited by small cohort sizes, heterogeneous sampling windows, and insufficient external validation.

Importantly, transcriptomic studies do not simply identify individual differentially expressed genes; rather, they provide a framework for understanding how inherited susceptibility is translated into dynamic immune programs during disease progression. These findings have contributed to the identification of candidate inflammatory, interferon-associated, and vascular-remodeling programs that may underlie clinical heterogeneity in KD. A comparison of previous omics-related reviews and the present framework is provided in Table 2.

**Table 2:** Comparison of previous kawasaki disease omics-related reviews and the present framework.

Review	Omics coverage	Clinical endpoints discussed	Temporal biology	External validation	Evidence for causal interpretation	Molecular stratification/endotyping
Kuo et al. (2015): Kawasaki disease: an update on genetic susceptibility and pathogenesis	Genomics, GWAS, immune-regulatory pathways	Disease susceptibility; limited discussion of coronary complications and treatment response	Not a major focus; mainly addresses inherited susceptibility	Population replication discussed; clinical validation limited	Genetic associations provide susceptibility evidence; functional mechanisms require further validation	Not evaluated
Dietz et al. (2017): Dissecting Kawasaki disease: a state-of-the-art review	Immunology, cytokine pathways, cellular immune mechanisms	Pathogenesis, inflammation, vascular injury, treatment mechanisms	Limited; mainly describes acute inflammatory processes	Limited	Functional immunological evidence; systems-level causality remains unclear	Not evaluated
Transcriptomic biomarker-focused reviews	Bulk transcriptomics, gene-expression signatures	Diagnosis, IVIG response, coronary-risk assessment	Limited; mainly single-time-point studies	Limited external validation	Association-based evidence; functional validation required	Not evaluated
Single-cell and cellular heterogeneity reviews	scRNA-seq, single-cell approaches	Immune-cell states and cellular mechanisms	Emerging; longitudinal datasets remain limited	Limited	Cellular states require functional validation	Exploratory cellular states
Microbiome-focused reviews	Microbiome sequencing, metagenomics	Disease association and host–microbiome interactions	Emerging; phase-dependent changes increasingly recognized	Limited due to geographic and methodological variation	Mainly associative evidence; causal relationships remain uncertain	Not evaluated
Multi-omics integration reviews	Genomics, transcriptomics, proteomics, metabolomics, microbiome, single-cell approaches	Molecular mechanisms and candidate biomarkers	Emerging but incompletely defined	Limited	Cross-platform convergence supports biological coherence but does not establish causality	Conceptual frameworks rather than validated molecular categories
Present framework: Multi-Omics Insights into Immune Dysregulation and Candidate Biological Programs in Kawasaki Disease	Genomics, epigenomics, transcriptomics, single-cell sequencing, proteomics, metabolomics, microbiome profiling	Diagnosis, IVIG-response prediction, coronary-risk stratification, precision immunomodulation	Explicitly considers dynamic biological trajectories	Emphasizes multicenter external validation and prospective studies	Distinguishes potential causal drivers from downstream consequences	Proposes candidate biological programs as an interpretative framework rather than validated molecular subtypes

Previous reviews have substantially advanced understanding of Kawasaki disease genetics, immune mechanisms, and individual omics-based findings. However, systematic integration of temporal biology, biomarker validation requirements, causal interpretation, and molecular stratification remains limited. The present framework integrates multi-omics evidence as interconnected biological programs and discusses their potential translation toward precision immunomodulation.

**Table 3:** Major GWAS-identified susceptibility loci in kawasaki disease.

Gene/locus	Pathway	Evidence strength	Clinical interpretation	Representative evidence
ITPKC	Ca^2^⁺–NFAT signaling and T-cell activation	Moderate—functional variant with replicated association	Supports Ca^2^⁺–NFAT and inflammasome biology; insufficient for individual prediction	(19, 25, 51)
CASP3	Apoptosis and inflammatory-cell turnover	Moderate—functional and candidate-association evidence	Supports apoptosis/NFAT regulation; clinical effect estimates remain inconsistent	(19, 51)
FCGR2A	Fc*γ*-receptor signaling and immune-complex handling	High—replicated genome-wide association	Robust susceptibility locus; IVIG-response and coronary utility remain unproven	(20, 52)
BLK	B-cell receptor signaling	High—replicated GWAS-region evidence	Supports B-cell biology; individual effect size is modest	(21, 25, 50, 51)
HLA region	Antigen presentation and T-cell recognition	Moderate—ancestry-dependent HLA associations	Supports antigen-presentation heterogeneity; not an individual diagnostic marker	(25, 51, 53)
CD40	T–B-cell co-stimulation	Moderate–high—GWAS-supported association	Supports T–B-cell co-stimulation; effect varies across ancestry groups	(50, 51)

Genetic associations can vary by ancestry and study design. The listed loci are susceptibility factors, not deterministic diagnostic markers.

GWAS, genome-wide association study; NFAT, nuclear factor of activated T cells; IVIG, intravenous immunoglobulin.

### 3.2 Bulk transcriptomic signatures

The first large-scale transcriptomic studies of KD demonstrated extensive alterations in peripheral blood gene expression during the acute inflammatory phase, revealing coordinated molecular responses rather than isolated changes in individual genes. Popper et al. performed one of the earliest gene-expression studies and identified reproducible transcriptional patterns associated with immune activation and inflammatory responses in KD (22).

Among the most consistently observed transcriptomic features is activation of myeloid inflammatory programs. Neutrophil-associated genes, including S100A8, S100A9, FCGR3B, CSF3R, MMP8, and MMP9, are repeatedly increased across independent transcriptomic datasets, highlighting enhanced granulocyte activation and inflammatory tissue-remodeling responses during acute disease (22, 24). The S100A8/S100A9 complex (calprotectin) functions as an endogenous danger-associated molecular pattern (DAMP) and may amplify inflammation through innate immune sensing pathways (28, 29). Rather than representing isolated biomarkers, these genes collectively reflect a broader neutrophil–monocyte inflammatory program that appears to be a major molecular feature of acute KD.

A second recurrent transcriptomic pattern involves activation of interferon-associated responses. KD patients frequently exhibit increased expression of interferon-stimulated genes (ISGs), including IFIT1, IFIT2, IFIT3, ISG15, MX1, and IFITM1/2/3 (23, 24). Although interferon signaling may reflect antiviral-like immune activation, its disease specificity and temporal stability remain uncertain. Therefore, interferon-related signatures should currently be interpreted as indicators of an immune activation state rather than disease-specific molecular markers.

In addition to neutrophil and interferon-associated programs, transcriptomic and functional studies support the involvement of inflammasome-related and NF-*κ*B-mediated inflammatory pathways in KD. Inflammasome-associated signaling contributes to IL-1-related inflammatory amplification (32), while NF-*κ*B activation has been demonstrated in peripheral monocytes/macrophages and T cells during acute disease (33). Together, these findings suggest that bulk transcriptomics provides evidence for interconnected inflammatory programs involving innate immune activation, cytokine amplification, and vascular injury responses.

### 3.3 Transcriptomic signatures associated with coronary artery complications

The development of coronary artery aneurysms represents the most severe vascular complication of KD and likely reflects the interaction between systemic inflammation and local vascular susceptibility. Transcriptomic studies suggest that patients with coronary complications exhibit enhanced activation of pathways involved in endothelial dysfunction, extracellular-matrix degradation, platelet activation, and vascular remodeling (40, 55).

Among these pathways, matrix metalloproteinases represent a key biological link between immune activation and vascular injury. Increased MMP-related transcriptional activity, particularly involving MMP9, has been associated with extracellular-matrix degradation and arterial wall remodeling in KD (36, 38, 39).

However, circulating or blood-derived transcriptomic signatures may not fully represent molecular events occurring within coronary arterial tissues. Therefore, vascular-remodeling signatures should be considered candidate indicators of coronary injury risk rather than validated predictive biomarkers.

Future studies integrating transcriptomic profiles with vascular imaging, proteomic markers, and longitudinal coronary outcomes may help define molecular patterns associated with coronary complications.

### 3.4 Single-cell RNA sequencing resolves cellular sources of immune programs

Single-cell RNA sequencing (scRNA-seq) provides a higher-resolution approach to characterize immune-cell composition, cellular states, receptor repertoires, and potential cell–cell interactions in KD.

Early single-cell studies based on peripheral blood mononuclear cells (PBMCs) primarily identified alterations in monocyte, B-cell, and T-cell populations. One of the earliest studies demonstrated strong monocyte transcriptional changes, reduced MHC class II expression, altered CD8+ T-cell composition, and post-IVIG plasma-cell expansion; however, the discovery cohort was limited by small sample size, including only seven patients and three healthy controls. Importantly, conventional PBMC-based approaches underrepresent mature neutrophils, which are increasingly recognized as major contributors to KD inflammation. Therefore, neutrophil-associated states should be evaluated using whole-blood, granulocyte-preserving approaches, functional studies, or dedicated single-cell protocols rather than inferred solely from PBMC datasets.

Subsequent single-cell analyses have further supported expansion and activation of inflammatory monocyte populations, including CD14+/FCN1-related states and S100A8/A9- and IL1B-associated programs (57, 58). These observations are consistent with bulk transcriptomic and protein-level evidence, suggesting that myeloid inflammatory activation represents a reproducible molecular feature of KD. Nevertheless, differences in cell annotation strategies, cohort sizes, disease stages, and treatment exposure currently limit the identification of stable disease-specific cellular states.

Although innate immune activation dominates the acute inflammatory landscape, adaptive immune responses also contribute to KD pathogenesis. Studies have identified activated CD4+ T cells, altered regulatory T-cell populations, and cytotoxic T-cell responses, supporting the involvement of both innate and adaptive immune networks (59, 60).

Emerging single-cell multi-omics approaches, including combined transcriptomic and chromatin accessibility profiling, provide opportunities to connect immune-cell states with regulatory mechanisms and identify regulatory elements underlying cellular heterogeneity (61). These approaches may help determine whether observed transcriptional programs represent stable cellular states or transient responses driven by inflammation and treatment exposure. Similar single-cell frameworks in inflammatory diseases have demonstrated the value of resolving transitional immune states and regulatory networks beyond conventional bulk transcriptomic analyses. Nevertheless, current KD single-cell datasets remain exploratory because of limited sample size, cohort diversity, and the lack of longitudinal validation.

### 3.5 Emerging biological programs and candidate molecular frameworks

Accumulating evidence indicates that clinically defined KD contains heterogeneous inflammatory trajectories; however, current studies have not established reproducible, mutually exclusive molecular subtypes (26, 34). Rather than representing discrete disease categories, current multi-omics evidence synthesis suggests the presence of overlapping biological programs that may contribute to clinical heterogeneity in KD. Importantly, these programs are not derived from a unified multi-omics clustering analysis or formally defined molecular classification system, but represent conceptual integrations supported by convergent findings across transcriptomic, proteomic, genetic, epigenomic, metabolomic, and single-cell studies.

Based on the consistency and biological relevance of reported molecular alterations, three candidate biological programs can be proposed as research frameworks for interpreting KD heterogeneity: These programs should not be considered equivalent biological categories. Rather, they represent different layers of the KD disease process, including upstream immune-regulatory states, active inflammatory responses, and downstream tissue-remodeling consequences.

1. Myeloid inflammatory activation program

The myeloid inflammatory program represents the most consistently observed biological pattern across KD multi-omics studies. It is supported by convergent transcriptomic, proteomic, and functional evidence involving S100A8/A9, IL1B, NLRP3-related signaling, neutrophil activation, and monocyte inflammatory responses. This program reflects an innate immune activation state characterized by enhanced inflammatory mediator production, danger-associated molecular pattern signaling, and immune-cell recruitment. However, the presence of this program should not be interpreted as a disease-specific molecular subtype because similar inflammatory signatures may occur in infectious and other systemic inflammatory conditions.

(1) Interferon-associated immune response program

The interferon-associated program is mainly supported by transcriptomic studies demonstrating increased expression of interferon-stimulated genes, including IFIT family members, ISG15, MX1, and CXCL10. This program reflects an interferon-enriched immune state that may indicate antiviral-like immune activation or broader inflammatory regulation. However, its specificity for KD, temporal stability during disease evolution, and relationship with other inflammatory programs remain uncertain. Therefore, interferon-associated signatures should currently be considered indicators of immune-state variation rather than independent molecular classifications.

(1) Vascular injury and remodeling response program: a downstream tissue-response program

The vascular injury and remodeling response represents a downstream tissue-response program rather than a classical immune activation state. Supported by transcriptomic, proteomic, and vascular biology studies, this program involves MMP9, VEGFA, endothelial activation markers, and extracellular-matrix regulation. Unlike myeloid and interferon-associated programs that primarily describe immune activation states, this program reflects downstream consequences of immune-mediated vascular injury and tissue remodeling. Therefore, vascular-remodeling signals may represent an outcome-associated biological response rather than an independent immune endotype.

Together, these overlapping programs provide a conceptual framework for understanding molecular heterogeneity in KD. These programs therefore operate at different biological levels, with inflammatory and interferon-associated programs reflecting immune activation states and vascular remodeling representing a downstream tissue-response consequence. They should be interpreted as evidence-based biological models rather than validated clinical endotypes, diagnostic classifications, or treatment-selection categories. Future studies integrating longitudinal multi-omics profiling, standardized clinical phenotyping, and therapeutic response data are required to determine whether these programs represent sequential or partially overlapping states along a shared inflammatory trajectory, rather than independent molecular subtypes. In addition to these candidate programs, upstream genetic and immune-regulatory pathways contribute to the threshold and intensity of immune activation in KD. For example, Ca^2^⁺–NFAT signaling, Fc*γ*-receptor biology, HLA-mediated antigen presentation, and CD40-related immune regulation may influence susceptibility and immune responsiveness but do not necessarily represent independent downstream molecular programs. Similarly, IL-6 signaling and neutrophil activation represent important inflammatory pathways that intersect with the proposed programs rather than constituting separate categories. Metabolic and microbiome-derived signals should also be considered as modulators of these biological states. Alterations in lipid metabolism, bile-acid pathways, and microbial metabolites may influence immune-cell activation, inflammatory persistence, and vascular responses through host–microbe interactions. However, current evidence remains insufficient to assign these factors to a single immune program, and future integrative studies are required to determine their position within KD inflammatory trajectories.

## 4 Epigenomic regulation and non-coding rnas shape immune responses in Kawasaki disease

Epigenomic alterations provide a potential regulatory link between inherited susceptibility, inflammatory exposure, cellular composition, and dynamic immune responses in Kawasaki disease (KD). Unlike genetic variants that represent relatively stable susceptibility factors, epigenetic modifications may reflect disease-phase-dependent immune remodeling and contribute to the regulation of inflammatory programs.

Blood methylation-array studies have identified widespread epigenetic alterations during acute KD, including differentially methylated loci and coordinated methylation–expression changes involving immune-related pathways. One study involving 24 KD cases and 24 controls, together with an independent expression-validation cohort, suggested a potential role for *β*-catenin-related regulation; however, interpretation remains limited by modest sample size, whole-blood analysis, and potential confounding from leukocyte composition and reverse causation (62).

Promoter-level studies have also linked methylation changes in immune-related genes, including FCGR2A, with KD susceptibility and IVIG response (63). However, these findings do not establish stable epigenetic biomarkers, as acute inflammation, age, immune-cell proportions, and treatment exposure may substantially influence epigenomic profiles. Longitudinal studies incorporating paired pretreatment, post-treatment, and convalescent samples with cell-type-aware analysis are therefore required to distinguish disease-associated epigenetic remodeling from transient inflammatory changes.

Non-coding RNA studies have identified altered circulating microRNAs (miRNAs), long non-coding RNAs (lncRNAs), and circular RNAs (circRNAs) in KD, suggesting additional regulatory layers controlling inflammatory activation and vascular responses. Among these candidates, miR-223 is one of the more consistently investigated molecules, while endothelial- and inflammation-associated miRNAs, including miR-126, miR-21, miR-145, and miR-155, provide mechanistic hypotheses related to immune regulation and vascular injury. Although meta-analyses suggest potential diagnostic value, current findings remain heterogeneous due to differences in sample sources, normalization strategies, disease stages, and control populations (46, 47).

The biological relevance of miR-223 is supported by its established roles in granulocyte function and vascular-cell regulation; however, mechanistic involvement alone does not establish disease specificity or clinical utility (64).

Future studies should integrate epigenomic, non-coding RNA, transcriptomic, and single-cell chromatin accessibility datasets to clarify how regulatory alterations shape immune-cell states and contribute to candidate biological programs in KD. Integrated single-cell transcriptomic and chromatin accessibility approaches have enabled direct linkage of cellular states with regulatory elements and may provide a framework for understanding immune-cell heterogeneity (61, 65). Single-cell chromatin accessibility studies have further demonstrated the potential of regulatory landscape profiling to identify cell-state-specific regulatory programs (66).

Therefore, epigenetic and non-coding RNA findings should currently be considered mechanistic indicators of immune-state remodeling rather than validated clinical biomarkers. Their potential contribution to molecular stratification and precision immunomodulation requires further evaluation in longitudinal, multicenter cohorts using clinically relevant febrile controls.

## 5 Proteomic, metabolic, and microbiome evidence reflect functional outputs of immune programs in Kawasaki disease

### 5.1 Proteomics and soluble immune mediators

Proteomic studies complement transcriptomic analyses by providing information on functional molecular outputs, including secreted inflammatory proteins, complement components, coagulation factors, and tissue-injury-related mediators. Compared with transcriptomic alterations, protein-level measurements may more directly reflect circulating inflammatory states and clinical phenotypes.

iTRAQ-based serum profiling has identified several candidate proteins, including S100A8, S100A9, S100A12, DEFA1, PRDX2, and ORM1, that may distinguish KD from febrile controls. Functional studies further suggest that selected proteins participate in neutrophil–endothelial interactions, supporting their potential involvement in inflammatory vascular injury (30). Serum exosome proteomic analyses have additionally implicated complement- and coagulation-related proteins, including FGG and C1QC, although current studies remain limited by small discovery cohorts, heterogeneous platforms, and insufficient external validation (41).

Recent KD proteomic studies have identified inflammatory, complement-related, and coagulation-associated protein networks; however, current evidence remains limited by small cohorts and insufficient multicenter validation (30, 41).

Importantly, protein abundance cannot be directly equated with corresponding RNA expression. Post-transcriptional regulation, protein secretion, cellular turnover, and technical factors may result in weak transcript–protein correlations. Therefore, proteomic candidates require validation in clinically relevant febrile control populations and should demonstrate added value beyond established clinical indicators, including CRP, NT-proBNP, albumin, and routine hematological parameters.

### 5.2 Metabolomics and lipidomics reveal immunometabolic alterations

Metabolomic and lipidomic studies provide additional insight into the metabolic consequences of immune activation in KD. Acute KD has been associated with alterations in lipid metabolism, amino-acid metabolism, and bile-acid pathways, suggesting that inflammatory responses are accompanied by systemic metabolic remodeling. Recent metabolomic and lipidomic studies suggest that immune activation in KD is accompanied by broader metabolic remodeling involving lipid mediators, bile-acid pathways, and energy metabolism. Integration of metabolomic data with transcriptomic, proteomic, and cytokine profiles may provide additional insight into inflammatory trajectories rather than isolated diagnostic markers. These metabolic alterations should therefore be interpreted as components of broader immunometabolic states that interact with inflammatory signaling, cellular activation, and vascular responses rather than as independent disease-specific biomarkers (42, 48). This concept is consistent with the emerging field of immunometabolism, in which metabolic states actively regulate immune-cell function and inflammatory responses (67).

A recent mass-spectrometry-based metabolomic study involving 108 KD patients and 52 non-KD controls identified altered bile-acid and lipid metabolic profiles and explored their associations with IVIG response and coronary injury (42). Other studies have reported associations between lipid-related parameters, including HDL-C, LDL-C, total cholesterol, and apolipoprotein A, with IVIG resistance or coronary complications (48). These associations require validation in larger prospective cohorts with standardized sampling and clinical phenotyping.

However, metabolic signatures are highly influenced by fasting status, diet, age, medication exposure, disease duration, liver function, and sample processing. Therefore, metabolomics is unlikely to provide an isolated diagnostic solution but may be particularly valuable when integrated with transcriptomic, proteomic, and cytokine data to characterize immunometabolic states associated with different KD inflammatory trajectories.

### 5.3 Microbiome and host–microbe interactions

Microbiome studies provide a potential link between environmental exposure and immune activation in genetically susceptible children. Longitudinal metagenomic analysis has identified phase-dependent alterations in microbial composition and enrichment of specific bacterial taxa, including Streptococcus species, during acute KD (68). More recent studies have reported reduced short-chain-fatty-acid-producing taxa and associations between microbial alterations and coronary artery lesions (69). Recent microbiome research has increasingly shifted from describing microbial composition alone toward investigating functional consequences, including microbial metabolites, short-chain fatty acids, bile-acid transformation, and host immune regulation. Such host–microbiome interactions represent a broader framework linking microbial signals with immune homeostasis and inflammation (70).

Nevertheless, current microbiome findings remain largely associative. Antibiotic exposure, diet, age, geographic variation, hospitalization, and acute inflammation can substantially influence microbial composition, and reverse causation cannot be excluded. To clarify whether microbiome alterations represent triggers, consequences, or modifiers of KD inflammation, future studies should incorporate pretreatment sampling, detailed antibiotic information, appropriate febrile controls, and integration with host immune and metabolic profiles. Importantly, microbiome alterations identified in KD should not be interpreted uniformly as causal mechanisms. Current evidence supports three possible biological interpretations: first, microbial components or metabolites may act as upstream environmental signals that modulate immune activation in genetically susceptible individuals; second, dysbiosis may represent a secondary consequence of systemic inflammation, hospitalization, dietary changes, or antibiotic exposure; and third, microbiome-derived signals may function as disease modifiers by shaping the magnitude or persistence of host immune responses. Therefore, distinguishing causal host–microbiome interactions from disease-associated dysbiosis requires longitudinal studies incorporating pretreatment sampling, functional validation, and integrated analysis of microbial composition, metabolites, and host immune responses.

### 5.4 Defining meaningful multi-omics integration in Kawasaki disease

The concept of “multi-omics” should extend beyond the parallel description of molecular alterations from different platforms. More informative approaches integrate multiple biological layers within the same individuals and determine whether cross-platform signals converge into reproducible immune programs.

In KD, meaningful integration may include genotype–expression relationships, methylation–transcriptome interactions, transcriptome–proteome concordance, and single-cell transcriptomic–chromatin accessibility analyses. Importantly, high diagnostic performance from discovery datasets alone is insufficient. Clinically relevant molecular signatures should demonstrate reproducibility, biological coherence, incremental value beyond established clinical variables, and potential impact on clinical decision-making.

### 5.5 Sources of heterogeneity in KD multi-omics studies

Despite substantial advances in multi-omics profiling, heterogeneity across KD studies remains a major barrier to identifying stable molecular signatures and clinically applicable biomarkers. Such heterogeneity arises from both biological variation among patients and methodological differences among studies.

Biological heterogeneity is particularly important in KD because molecular profiles dynamically change during disease evolution. Disease day at sampling, inflammatory severity, age, genetic background, ancestry, and ethnicity-related population differences may influence immune-cell composition, cytokine production, gene-expression patterns, and metabolic states. For example, younger patients and patients sampled during different inflammatory phases may exhibit distinct transcriptomic and proteomic profiles despite having the same clinical diagnosis. Therefore, molecular signatures identified from heterogeneous cohorts may reflect differences in disease timing rather than true biological subtypes.

Treatment-related factors represent another major source of variation. IVIG exposure, corticosteroids, biologic agents, and antibiotic administration may substantially alter immune activation states, circulating cytokine concentrations, leukocyte phenotypes, microbiome composition, and metabolic profiles. Pretreatment sampling is therefore essential for biomarker discovery aimed at diagnosis or prediction of initial IVIG response. Post-treatment molecular changes may provide valuable information regarding treatment effects or prognosis but should not be interpreted as pretreatment predictive signatures.

Cellular composition is a critical confounder in blood-based multi-omics analyses. Bulk transcriptomic, epigenomic, and proteomic measurements may reflect differences in circulating neutrophils, monocytes, lymphocytes, and other immune-cell populations rather than intrinsic changes within specific cell types. Single-cell approaches and computational deconvolution methods can partially address this limitation; however, differences in cell annotation strategies and analytical pipelines remain important sources of variability.

Technical heterogeneity also contributes to inconsistent findings across studies, including differences in sequencing platforms, assay sensitivity, sample processing, normalization methods, and statistical approaches. Microbiome studies are particularly affected by geographic variation, diet, antibiotic exposure, and sequencing methodology, whereas metabolomic analyses are sensitive to fasting status, medication exposure, and pre-analytical handling.

Addressing these sources of heterogeneity requires standardized pretreatment sampling, detailed clinical annotation, adjustment for cellular composition, harmonized outcome definitions, and multicenter external validation. Future KD multi-omics studies should integrate biological and technical variables into analytical models rather than treating molecular signatures as independent disease features. Such approaches may help distinguish true disease-associated biological programs from signatures driven by sampling differences, treatment effects, or technical variation.

## 6 Clinical translation of multi-omics biomarkers: from discovery to decision-making

### 6.1 Translation begins with the clinical decision

Biomarker development in KD should begin with a clearly defined clinical decision rather than the availability of high-dimensional molecular measurements. The principal use cases are: distinguishing KD from competing febrile illnesses, identifying likely IVIG non-response before initial treatment, and estimating the risk of coronary artery abnormalities. These tasks have different comparators, sampling windows, acceptable error rates, and clinical consequences; a marker optimized for one should not be assumed to perform another. Conventional variables—including age, illness day, CRP, albumin, sodium, platelet count, alanine aminotransferase, and echocardiographic findings—must form the reference model against which omics adds value (2, 14, 16–18, 56, 71).

A critical distinction in biomarker translation is the difference between prognostic and predictive biomarkers. Prognostic biomarkers provide information about the likelihood of future clinical outcomes independent of a specific intervention, such as the risk of coronary artery complications or persistent inflammation. In contrast, predictive biomarkers identify patients who are more likely to benefit from a particular treatment strategy and require evidence of treatment-by-biomarker interaction. In KD, many reported molecular signatures, including inflammatory, vascular, and metabolic profiles, currently have prognostic or risk-stratification implications but should not be interpreted as predictive biomarkers for treatment selection without prospective interventional validation.

### 6.2 KD diagnosis vs. febrile controls

Healthy-control comparisons exaggerate apparent specificity because they primarily detect inflammation. The appropriate diagnostic population comprises children evaluated for KD before IVIG, including bacterial and viral infections, systemic inflammatory disorders, and other causes of prolonged fever. Recurrent signals such as S100A8/A9, FCGR3B, CSF3R, MMP8, interferon-stimulated genes, and selected protein panels are biologically credible candidate diagnostic biomarkers (22–24, 28–31). However, the biological plausibility of these markers does not necessarily indicate KD-specific diagnostic specificity. Many inflammatory signatures identified by multi-omics studies reflect shared host-response programs rather than disease-exclusive mechanisms. For example, S100A8/A9 and neutrophil-associated genes may indicate innate immune activation but can also be elevated in bacterial infections, severe viral infections, and other inflammatory disorders. Similarly, interferon-stimulated gene signatures may reflect antiviral immune activation rather than KD-specific biology. Therefore, candidate biomarkers should be evaluated against clinically relevant febrile controls rather than healthy children alone, and their clinical value should be determined by whether they improve diagnostic discrimination beyond routine clinical parameters. However, S100A8/A9 and neutrophil activation also occur in infection and sterile inflammation, whereas interferon-rich programs may reflect viral exposure. Their likely clinical value lies not in stand-alone specificity, but in integrated biomarker combinations that capture KD-associated patterns of immune activation, vascular involvement, and disease evolution while improving discrimination from competing febrile illnesses.

Diagnostic studies should report complete and incomplete KD separately, define illness day and pretreatment status, use adjudicated final diagnoses, and retain indeterminate cases. Sensitivity at clinically acceptable false-positive rates is more informative than a discovery AUC alone. A useful test must shorten diagnostic delay or improve treatment decisions beyond clinical criteria and echocardiography.

### 6.3 IVIG resistance prediction

Approximately 10%–20% of patients have persistent or recurrent fever after initial IVIG (14, 15, 71). Studies associate non-response with heightened IL-1, TNF, and NF-κB signaling, S100A8/A9-rich neutrophil activation, complement/coagulation changes, and altered lipid or bile-acid metabolism (30, 32, 33, 41, 42, 48). These observations support an inflammatory-burden model of resistance, but they do not establish a validated molecular classifier. Existing clinical scores also show limited transportability across ancestry groups (16–18).

A translational model should use samples obtained before initial IVIG, apply a prespecified definition of response, and evaluate incremental value over a locally calibrated clinical score. Because the intended action may be adjunctive corticosteroid, infliximab, IL-1 blockade, or intensified monitoring, false-positive escalation and treatment toxicity must be incorporated into utility analyses. Most importantly, association with IVIG resistance is not equivalent to predicting benefit from a specific adjunctive agent; that claim requires a treatment-by-signature interaction in a prospective trial.

### 6.4 Coronary risk stratification

Coronary outcomes in KD reflect more than systemic inflammatory intensity. Endothelial activation, vascular permeability, leukocyte adhesion, extracellular-matrix degradation, and smooth-muscle-cell remodeling connect acute immune injury to aneurysm development (36–40, 45, 55). MMP9, VEGFA, ICAM1/VCAM1, complement–coagulation proteins, and lipid-related measures are therefore plausible components of a coronary-risk signature, but none is a validated stand-alone predictor.

Studies should use harmonized coronary Z-score definitions, distinguish transient dilation from persistent aneurysm, account for baseline coronary status, and include serial echocardiography. A molecular test is most credible if it improves early surveillance intensity or therapeutic decisions before coronary changes become apparent. Models that include post-treatment measurements may be useful for prognosis but must not be presented as pretreatment prediction tools.

Interpretation of molecular predictors of coronary complications is further complicated by heterogeneity in coronary outcome definitions across studies. Reported endpoints have included coronary artery dilation, coronary artery lesions, and coronary artery aneurysms, with different thresholds and follow-up periods applied among cohorts. These definitions may represent partially overlapping but biologically distinct stages of vascular involvement, ranging from early transient vascular responses to persistent structural remodeling. In addition, differences in echocardiographic timing relative to disease onset, IVIG administration, and follow-up duration may influence observed associations between molecular signatures and coronary outcomes. Therefore, future multi-omics biomarker studies should incorporate standardized coronary outcome definitions, harmonized Z-score criteria, and longitudinal clinical follow-up to ensure meaningful comparison across cohorts.

### 6.5 Requirements for clinical implementation

Four requirements separate discovery from implementation. First, the assay must be analytically reproducible, with standardized collection, processing, normalization, quality control, and reporting. Second, clinical validation must use locked features, coefficients, and thresholds in multicenter external cohorts that reflect the intended population and ancestry mix. Third, reporting must include calibration, clinically relevant sensitivity and specificity, missing-data handling, and incremental performance over a clinical baseline; decision-curve analysis is preferable to AUC-only claims. Fourth, clinical utility must be demonstrated prospectively by showing that test-guided care changes management or outcomes without unacceptable overtreatment.

High-dimensional machine-learning models are especially vulnerable to leakage, overfitting, and optimistic internal validation (72). Explainability methods such as SHAP describe how a model generated predictions; they do not establish causality, transportability, or clinical benefit. Such panels should ideally capture complementary immune programs rather than isolated biomarkers. Recent advances in high-throughput profiling and computational integration have expanded opportunities for biomarker discovery; however, translation into clinical practice requires prospective validation in clinically relevant populations and demonstration of added value beyond established clinical parameters.

## 7 Precision immunomodulation framework and future perspectives

### 7.1 Candidate biological programs as a framework for future molecular stratification

Clinical KD likely represents a spectrum of inflammatory trajectories rather than a single biologically uniform disease entity. Although current genomic, transcriptomic, proteomic, and single-cell studies have not established reproducible molecular subtypes, accumulating evidence suggests the presence of interconnected biological programs that may contribute to clinical heterogeneity in KD. Importantly, these programs should not be interpreted as independent or mutually exclusive molecular endotypes, but rather as dynamic components of a complex immune–vascular response influenced by disease phase, cellular composition, genetic susceptibility, and treatment exposure (26, 34).

Across multi-omics studies, several recurring biological programs have been observed, including inflammatory activation states, interferon-associated immune responses, and vascular injury/remodeling responses. These programs represent interconnected components of KD pathobiology rather than independent molecular endotypes. The inflammatory activation program is characterized by IL-1/NLRP3 signaling, S100A8/A9-associated neutrophil–monocyte activation, and enhanced cytokine amplification, representing a major innate immune response state observed across transcriptomic and proteomic studies. The interferon-associated program, enriched for interferon-stimulated genes such as IFIT family members, ISG15, and MX1, reflects an immune activation state that may vary according to disease phase and host immune context. In contrast, vascular injury/remodeling represents a downstream tissue-response program involving endothelial activation, extracellular-matrix degradation, and vascular remodeling pathways, including VEGFA and MMP9-related mechanisms, rather than a classical immune activation state.

Upstream immune-regulatory pathways, including Ca^2^⁺–NFAT signaling, Fc*γ*-receptor-mediated immune-complex regulation, HLA-associated antigen presentation, and CD40-related immune interactions, may shape the threshold and magnitude of immune activation in genetically susceptible individuals. These pathways contribute to disease susceptibility and immune responsiveness but should not be considered separate downstream molecular programs. Similarly, IL-6 signaling and neutrophil activation represent important inflammatory pathways that intersect with multiple biological programs rather than defining independent categories.

In addition to immune signaling pathways, metabolic and microbiome-derived factors may modulate these biological responses. Alterations in lipid metabolism, bile-acid pathways, and microbial metabolites may influence immune-cell activation, inflammatory persistence, and vascular responses through host–microbe interactions. However, current evidence remains insufficient to assign these factors to a specific biological program, and their contribution to KD immune trajectories requires further investigation through integrated multi-omics studies.

Therefore, the current value of multi-omics approaches may not lie in defining fixed molecular categories of KD, but in reconstructing dynamic biological states associated with disease evolution and clinical outcomes. Future longitudinal studies integrating multi-omics profiles, clinical phenotypes, coronary outcomes, and treatment responses will be required to determine whether these biological programs represent sequential phases along a shared inflammatory trajectory, partially overlapping immune states, or clinically actionable patterns for precision immunomodulation.

Table 4 summarizes these candidate immune programs and their current evidence maturity. These programs should currently be considered biological frameworks for research interpretation rather than validated tools for molecular stratification or treatment selection.

**Table 4:** Candidate biological programs and their translational implications in kawasaki disease.

Candidate biological program	Core molecular and cellular features	Evidence maturity	Testable translational hypothesis	Representative evidence
Innate inflammatory	IL1B/NLRP3; CXCL8; S100A8/A9; activated neutrophils and monocytes	Moderate—recurrent blood, cytokine, and mechanistic evidence	A locked IL-1/S100 panel may enrich prospective trials of inflammatory escalation	(11, 22–24, 28, 29, 32–34)
Interferon-rich	IFIT1/IFIT3; ISG15; MX1; IFITM-family genes	Low–moderate—reproducible in some cohorts but temporally variable	An interferon-rich signature may refine research stratification, not routine treatment selection	(22–24, 34)
Vascular injury/remodeling	VEGFA; MMP9; ICAM1/VCAM1; endothelial activation and ECM remodeling	Moderate mechanistic evidence; low prospective predictive maturity	A vascular-injury panel may add to serial echocardiography for coronary-risk estimation	(11, 34–40, 45, 55, 57)
Treatment-response-associated	Persistent IL-1/TNF/NF-κB activity; neutrophil activation; complement/coagulation and metabolic perturbation	Low—mostly retrospective association; treatment interactions untested	A pretreatment signature should be tested for interaction with adjunctive immunomodulation	(14–18, 30, 32, 42, 56, 71, 73–75)

These overlapping patterns synthesize published observations for research use. They are not prospectively validated, mutually exclusive clinical subtypes or treatment-selection rules.

ECM, extracellular matrix; ISG, interferon-stimulated gene; IL, interleukin.

### 7.2 Mechanism-directed immunomodulation and future therapeutic stratification

Mechanistic studies have identified several potentially actionable inflammatory pathways in KD, but molecularly guided treatment selection has not yet become part of routine clinical practice. A major challenge for precision immunomodulation is distinguishing causal biomarkers that participate in disease initiation or amplification from consequential biomarkers that reflect downstream effects of inflammation or tissue injury. Many molecular signatures identified by KD multi-omics studies, including inflammatory cytokines, acute-phase proteins, and vascular injury markers, may represent consequences of immune activation rather than independent drivers of disease. In contrast, upstream regulatory pathways involving genetic susceptibility, immune-cell activation thresholds, and specific inflammatory signaling networks may contribute more directly to disease pathogenesis. However, current evidence is insufficient to definitively classify most candidate biomarkers as causal or consequential. Associations identified by multi-omics approaches should therefore be interpreted within temporal and functional contexts, as statistical association alone does not establish biological causality. This distinction has important implications for precision immunomodulation. Biomarkers reflecting inflammatory burden may be useful for risk stratification or disease monitoring, whereas predictive biomarkers for treatment selection require evidence that modifying a specific pathway produces differential clinical benefit in biomarker-defined populations. Therefore, future studies should integrate longitudinal multi-omics data, functional experiments, and prospective treatment-response analyses to identify molecular features that are both biologically meaningful and therapeutically actionable. Current treatment decisions should continue to follow established guidelines and incorporate individual clinical characteristics and coronary risk assessment.

The IL-1 pathway represents one of the most biologically supported therapeutic targets. Endothelial IL-1 production, inflammasome activation, NF-*κ*B signaling, and IL1B-enriched myeloid programs suggest a coordinated inflammator*y* axis in KD (11, 23, 32, 33). S100A8/A9 may further amplify this pathway through danger signaling and neutrophil–monocyte interactions (28, 29). However, biological plausibility alone does not identify which patients are most likely to benefit from IL-1 blockade. Therefore, IL-1/S100-associated signatures represent potential predictive biomarkers that require prospective treatment-by-signature validation, rather than simply reflecting inflammatory activity.

Similarly, clinical studies of infliximab and other immunomodulatory agents provide evidence supporting pathway-directed therapy in selected KD populations (54, 74). However, these approaches do not yet represent omics-guided treatment strategies. Small studies exploring agents such as tocilizumab require further validation regarding efficacy, safety, and patient selection (75).

The future goal of precision immunomodulation is therefore not simply to identify stronger inflammatory markers, but to integrate pretreatment immune programs with clinical variables, disease stage, and coronary status to identify patients most likely to benefit from specific interventions. Such treatment–signature interactions require prospective clinical trials (73).

### 7.3 Computational integration and study design

Computational approaches, including machine-learning methods, provide opportunities to integrate clinical and molecular variables in KD. Recent advances in machine-learning-based multi-omics integration provide new opportunities to identify candidate molecular patterns associated with clinical outcomes. Multi-omics integration frameworks such as latent-factor models provide additional strategies for identifying coordinated molecular patterns across multiple biological layers (76). However, these approaches require rigorous external validation, avoidance of overfitting, and demonstration of clinical utility before translation into practice. However, small sample sizes, high-dimensional datasets, and limited external validation create substantial risks of overfitting and inflated performance estimates. Explainability approaches such as SHAP may clarify model behavior but cannot establish biological causality or clinical utility (72).

Beyond technical limitations, biological heterogeneity represents a fundamental challenge for defining stable molecular endotypes in KD. Molecular signatures identified in different cohorts may reflect distinct combinations of disease phase, inflammatory intensity, cellular composition, genetic background, and treatment exposure rather than reproducible disease categories. For example, a neutrophil-enriched transcriptomic signature may indicate acute inflammatory activation in one cohort but may primarily reflect differences in circulating cell proportions in another. Similarly, post-IVIG molecular profiles may capture treatment-induced immune modulation rather than pretreatment disease biology. Therefore, molecular programs identified by multi-omics approaches should be interpreted within their clinical and temporal context, and future endotype discovery studies should incorporate standardized sampling windows, cell-type-resolved measurements, longitudinal follow-up, and external validation across diverse populations.

Single-cell and spatial technologies may further resolve immune–vascular interactions; however, coronary tissue remains difficult to obtain, and circulating immune profiles may not fully represent vascular pathology. Current KD single-cell multi-omics studies remain exploratory and require validation in larger, clinically annotated cohorts (65).

Future international studies should prioritize standardized pretreatment sampling, harmonized coronary outcomes, longitudinal follow-up, and integration of multi-omics data with clinical decision models. Ultimately, the clinical value of computational approaches will depend not on model complexity, but on whether they can distinguish stable biological signals from disease-stage, treatment-related, and technical variation, thereby improving patient stratification and therapeutic decision-making beyond existing clinical criteria (77, 78).

### 7.4 Temporal multi-omics trajectories and precision risk stratification

KD is a dynamic inflammatory disease in which molecular signatures may change substantially across different phases of disease evolution. Therefore, the clinical value of multi-omics biomarkers depends not only on their biological identity but also on the timing of measurement. A single molecular snapshot may capture a transient inflammatory state rather than a stable disease feature. Longitudinal multi-omics approaches may therefore provide a more informative framework by reconstructing molecular trajectories from disease onset to treatment response and vascular outcomes.

During the early diagnostic phase, molecular signatures reflecting innate immune activation, including neutrophil–monocyte programs, IL-1/NF-*κ*B signaling, and inflammatory mediators, may help distinguish KD from other febrile illnesses. However, these signals require validation in pretreatment cohorts with clinically relevant febrile controls because many inflammatory responses are shared across infectious and inflammatory diseases.

For IVIG-response prediction, the most clinically relevant biomarkers should be obtained before initial therapy and evaluated according to predefined treatment outcomes. Molecular signatures associated with persistent inflammatory activation, including IL-1/TNF/NF-*κ*B pathways, complement–coagulation alterations, and immunometabolic changes, may identify patients at increased risk of inadequate response. However, association with IVIG resistance does not establish prediction of benefit from a specific adjunctive therapy, which requires prospective treatment-by-signature interaction studies.

For coronary risk assessment, molecular patterns associated with endothelial activation, extracellular-matrix remodeling, and vascular injury may become more informative when integrated with serial echocardiographic measurements and clinical variables. Post-treatment molecular profiles may provide prognostic information regarding ongoing inflammation or vascular injury but should be distinguished from pretreatment predictive biomarkers.

Machine-learning approaches may facilitate integration of longitudinal multi-omics data with clinical variables to identify dynamic risk trajectories. However, such models require adequate sample sizes, external validation, biological interpretation, and assessment of clinical utility before implementation. Ultimately, the goal of temporal multi-omics integration is not simply to identify the strongest biomarker at a single time point, but to define disease trajectories that support individualized risk assessment and precision immunomodulation. A conceptual model of these temporal molecular trajectories and their potential clinical decision points is illustrated in Figure 3.

**Figure 3:**
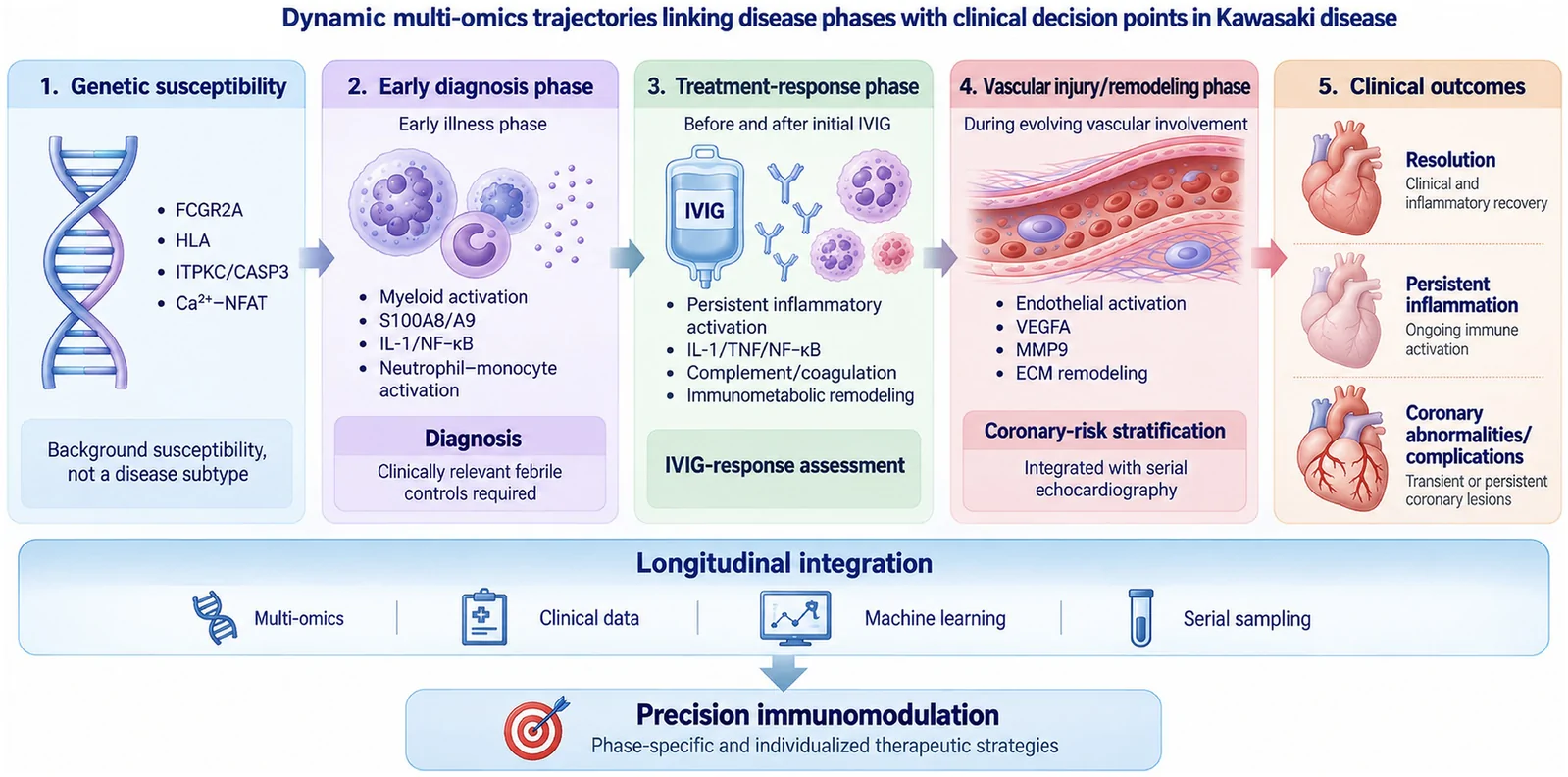
Conceptual framework of temporal multi-omics trajectories and clinical decision points in kawasaki disease. KD molecular signatures may change during disease evolution and should be interpreted within temporal and clinical contexts. Early inflammatory programs may contribute to diagnostic discrimination from febrile controls, whereas pretreatment immune signatures may help identify patients at risk of inadequate IVIG response. Vascular injury and remodeling signatures may provide prognostic information when integrated with serial coronary assessment. This framework represents a hypothesis-generating model for longitudinal multi-omics investigation rather than a validated disease staging system or established molecular trajectory. Longitudinal multi-omics integration, machine-learning approaches, and prospective treatment-response studies are required to determine whether these trajectories can support precision immunomodulation.

## 8 Conclusions

Kawasaki disease is a complex immune-mediated pediatric vasculitis in which genetic susceptibility interacts with dynamic immune activation, vascular injury, metabolic remodeling, microbiome-associated influences, and environmental factors. Integrative multi-omics studies have substantially expanded our understanding of KD pathobiology by revealing convergent biological processes involving Ca^2^⁺–NFAT and Fc*γ*-receptor signaling, myeloid inflammatory activation, IL-1/S100 pathways, interferon-associated responses, complement–coagulation regulation, endothelial dysfunction, and extracellular-matrix remodeling.

However, current evidence does not support the existence of universally reproducible or clinically actionable molecular subtypes. Instead, multi-omics-derived signatures are better interpreted as overlapping biological programs that may represent different immune states along a shared inflammatory trajectory. The observed molecular heterogeneity likely reflects a combination of disease phase, cellular composition, genetic background, ancestry, treatment exposure, and technical factors. Therefore, the primary contribution of multi-omics approaches may not be the classification of patients into fixed molecular categories, but the reconstruction of dynamic immune states associated with clinical heterogeneity and disease evolution.

Although numerous candidate biomarkers have been identified for KD diagnosis, IVIG-response assessment, and coronary risk stratification, most remain exploratory. Importantly, the diagnostic value of these signatures should be determined by their ability to discriminate KD from clinically relevant febrile illnesses rather than from healthy controls alone. Their clinical translation is limited by insufficient specificity against clinically relevant febrile controls, heterogeneous outcome definitions, limited external validation, and potential confounding from treatment and sampling timing. Future studies should prioritize standardized pretreatment sampling, longitudinal multi-omics profiling, harmonized coronary outcome assessment, and integration with clinical variables to establish robust and clinically applicable biomarker models.

The future of KD precision medicine will likely depend on identifying biologically meaningful molecular programs that can guide risk assessment and therapeutic stratification rather than searching for a single universal biomarker. Importantly, distinguishing prognostic biomarkers from predictive biomarkers will be essential for developing treatment strategies based on molecular characteristics. Prospective studies incorporating functional validation, machine-learning approaches, and treatment-by-signature interaction analyses are required to determine whether specific immune programs can identify patients who may benefit from targeted immunomodulatory therapies.

Ultimately, multi-omics approaches provide a framework for exploring the transition from phenotype-based classification toward molecularly informed immune stratification in KD. By integrating genetic susceptibility, dynamic immune responses, vascular injury pathways, microbiome influences, and clinical phenotypes, future research may enable more precise diagnosis, improved risk prediction, and individualized immunomodulation strategies for children with Kawasaki disease.
